# Supplementary material for: Ischemic Postconditioning Regulates New Cell Death Mechanisms in Stroke: Disulfidptosis
Source: Biomolecules. 2024 Oct 31;14(11):1390. doi: 10.3390/biom14111390 (PMC11591815; doi:10.3390/biom14111390)
Supplement: Supplementary file 1 [file biomolecules-14-01390-s001.zip › Supplementary figure notes S1-S2.pdf]

Supplementary figure notes

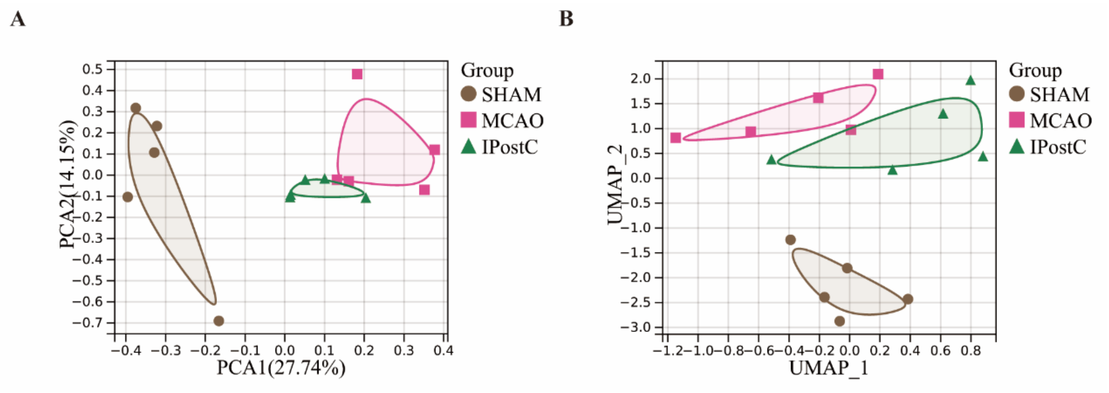

Figure S1. Multidimensional analysis of gene expression profiles. (A) Principal Component Analysis (PCA) displaying the separation of samples into three distinct groups: SHAM (brown), MCAo (green), and IPostC (pink). The percentage of variance explained by the principal components is indicated on the axes. (B) Uniform Manifold Approximation and Projection (UMAP) demonstrating the clustering of SHAM, MCAo, and IPostC samples, with distinct grouping patterns emerging in a two-dimensional space. Each symbol represents a sample, and the ellipses indicate the group dispersion within the plotted space.

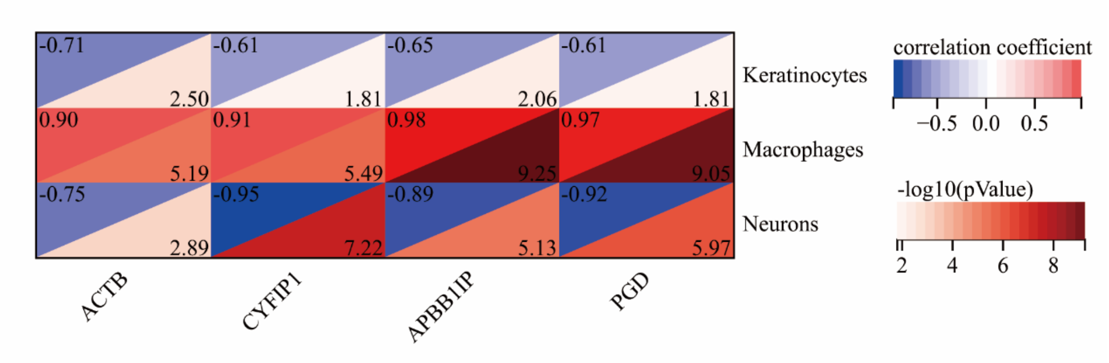

Figure S2. Correlation of central genes (ACTB, APBB1IP, CYFIP1 and PGD) with expression in three cell types (macrophages, keratinocytes and neurons). Each square represents a pairing of a gene and a cell type (column and row). Color indicates Spearman correlation coefficient (top left legend), while color intensity represents significance, expressed as -log10 (p-value) (bottom right). Red indicates positive correlations, blue indicates negative correlations, and stronger correlations (closer to 1 or -1) are represented by deeper colors. Lower p-values, indicating higher significance, are represented by deeper colors.
