# Supplementary figures and images for: Ischemic Postconditioning Regulates New Cell Death Mechanisms in Stroke: Disulfidptosis
Source: Biomolecules. 2024 Oct 31;14(11):1390. doi: 10.3390/biom14111390 (PMC11591815; doi:10.3390/biom14111390)

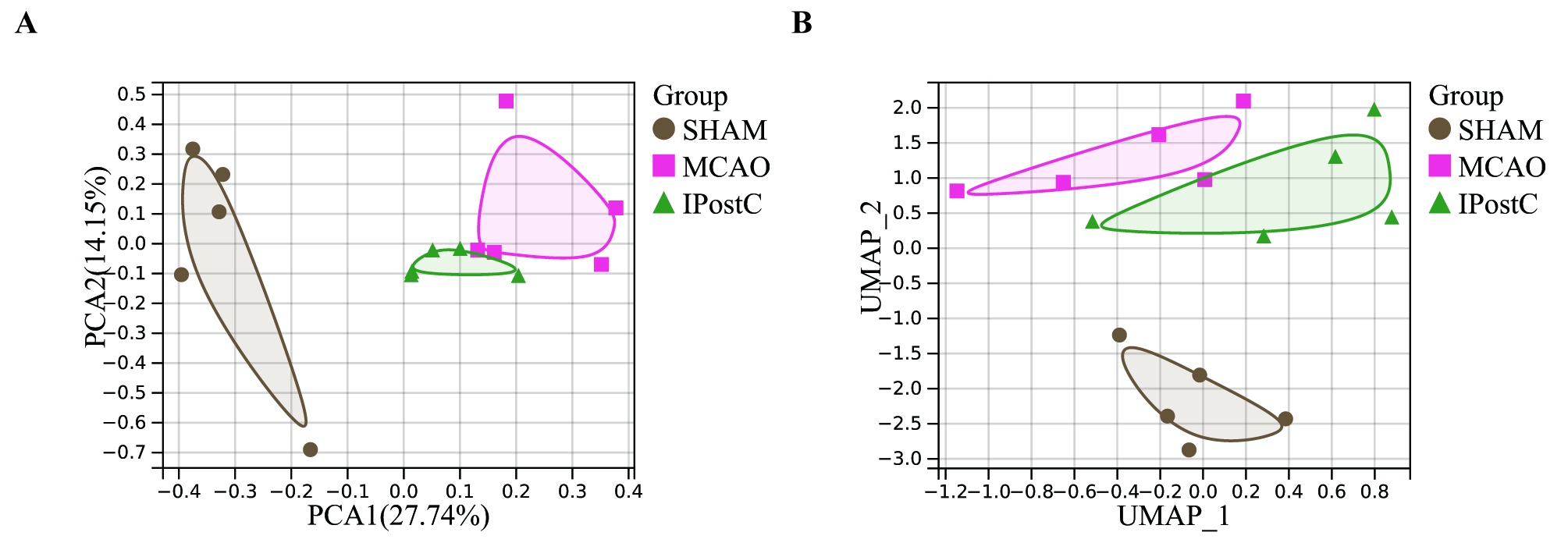

Supplement: Supplementary file 1 [file biomolecules-14-01390-s001.zip › Figure S1.tif]

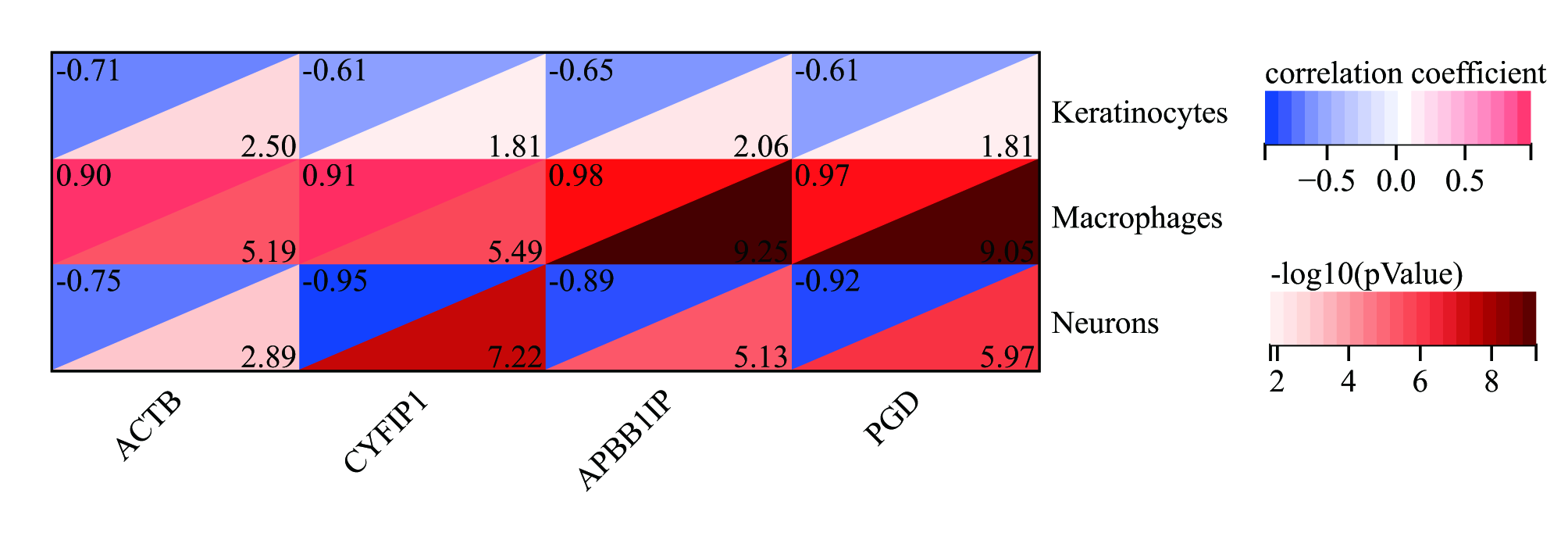

Supplement: Supplementary file 1 [file biomolecules-14-01390-s001.zip › Figure S2.tif]
